# The Presence of Calcified Raphe Is an Independent Predictor of Adverse Long-Term Clinical Outcomes in Patients With Bicuspid Aortic Stenosis Undergoing Transcatheter Aortic Valve Replacement

**DOI:** 10.3389/fcvm.2022.767906

**Published:** 2022-04-13

**Authors:** Yung-Tsai Lee, Wei-Hsian Yin, Tien-Ping Tsao, Kuo-Chen Lee, Ming-Chon Hsiung, Yun-Hsuan Tzeng, Jeng Wei

**Affiliations:** ^1^Heart Center, Cheng Hsin General Hospital, Taipei, Taiwan; ^2^Institute of Microbiology and Immunology, National Yang Ming Chiao Tung University, Taipei, Taiwan; ^3^Faculty of Medicine, School of Medicine, National Yang Ming Chiao Tung University, Taipei, Taiwan; ^4^Faculty of Medicine, National Defense Medical Center, Taipei, Taiwan; ^5^Division of Medical Imaging, Health Management Center, Cheng Hsin General Hospital, Taipei, Taiwan

**Keywords:** transcatheter aortic valve replacement, bicuspid aortic valve, aortic stenosis, balloon-expandable valve, valve calcification, calcified raphe, clinical outcomes

## Abstract

**Objective:**

Current guidelines recommend that transcatheter aortic valve replacement (TAVR) for bicuspid aortic valve (BAV) with aortic stenosis (AS) should only be performed in selected patients. However, we consider it even more crucial to identify what the really important factors are while determining long-term outcomes in patients with BAV undergoing TAVR, which is precisely the aim of this study.

**Methods:**

We retrospectively evaluated consecutive patients who underwent TAVR with balloon-expandable Sapien XT or Sapien 3 valves (Edwards Lifesciences, Irvine, CA) for the treatment of severe bicuspid AS. The primary end points were major adverse cardiac and cerebral events (MACCE), that is, mortality, non-fatal myocardial infarction (MI), disabling stroke, valve failure needing reintervention, or clinically relevant valve thrombosis during follow-up.

**Results:**

A total of 56 patients who underwent TAVR with Sapien XT (*n* = 20) or Sapien 3 (*n* = 36) were included. The device and procedural success rates were similar between the two TAVR valves; however, the newer-generation Sapien 3 yielded a trend toward better long-term clinical outcomes than the early-generation Sapien XT did (MACCE rates 35 vs. 11%, *p* = 0.071). In the multivariate Cox proportional hazards analyses, the presence of calcified raphe > 4 mm was the only independent predictor of long-term MACCE (hazard ratio: 6.76; 95% confidence interval: 1.21–37.67, *p* = 0.029).

**Conclusion:**

TAVR performed by a skilled heart team, while using newer-generation balloon-expandable Sapien 3 valve, may yield better long-term clinical outcomes compared to TAVR using early-generation Sapien XT valve. Moreover, the presence of calcified raphe >4 mm is an independent determinant of adverse clinical outcomes.

## Introduction

More often than not, bicuspid aortic valve (BAV) with aortic stenosis (AS) is congenital, whereas an acquired BAV occurs when there is a fibrous fusion between cusps of a preexisting tricuspid aortic valve (1, 2). Although there have existed sound enough data concerning the safety and efficacy of transcatheter aortic valve replacement (TAVR) in patients with tricuspid valve severe AS (3–5), patients with BAV have largely been excluded from randomized clinical trials involving TAVR (3–5). BAV consists of ~10% of patients currently treated by TAVR; however, despite encouraging data from registries, including patients with BAV who showed similar or even better outcomes of TAVR in bicuspid vs. tricuspid AS, TAVR has yet to establish itself in this patient cohort (6–15).

Because of the improvements in the design of sealing skirts of newer-generation transcatheter heart valves (THVs), procedural success has increased, and the survival rates of patients with BAV have become similar to those of patients with tricuspid valve AS undergoing TAVR (9–15). However, complications, such as moderate or severe paravalvular leakage (PVL) and aortic root dissection are more commonly seen in patients with BAV compared with those in patients with tricuspid aortic valve (9–15). Hence, certain experts proposed new BAV imaging classification for the patients who underwent TAVR and, to reduce complications, various supra-annular sizing methods, algorithms, balloon sizing, or even computer simulation to improve valve sizing and device selection (12, 16–26). However, whether these approaches can truly provide additional benefits in terms of improving either device or procedural success, or even clinical outcomes, remain controversial (26–29).

In the future, specifically designed prospective studies are required to provide further evidence on THV durability, anatomical selection criteria, and long-term success before TAVR can be established as a preferred option for patients with BAV. At this stage, we consider it more pressing to identify what the truly important factors are that determine the device success and long-term outcomes in patients with BAV undergoing TAVR; hence, it was established as the aim of this study.

## Materials and Methods

### Patient Population

From April 2016 to December 2020, a total of 56 consecutive patients with BAV disease and severe AS at intermediate or high risk for conventional cardiac surgery with sternotomy and cardiopulmonary bypass underwent TAVR with balloon-expandable valves in a high-volume center in Taiwan. They were referred to the TAVR multidisciplinary team composed of interventional cardiologists, imaging cardiologists, cardiothoracic surgeons, and anesthesiologists. This study was approved by the Institutional Review Board of Cheng Hsin General Hospital No. (769) 109A-09, and individual consent for this retrospective analysis was waived. In our institution, a shared decision-making approach is performed for all patients considering aortic valve replacement, with the implementation of best practices to ensure patient goals and preferences incorporated into final decision-making.

### Choice of Device, Vascular Access, and TAVR Procedures

The heart team of Cheng Hsin General Hospital is one of the largest and most experienced in Taiwan and proficient in doing TAVR with all available devices. The decisions whether TAVR may be performed or which type and size of the prosthesis to be used were subject to the heart team's discretion.

The TAVR procedure was first performed in Taiwan in 2010. The early valve technologies available were, mainly, the Medtronic CoreValve, Lotus (Boston Scientific, Natick, MA), and Sapien XT (Edwards Lifesciences, Irvine, CA), launched respectively in 2012, 2015, and 2016. Although there are no data indicating any one TAVR device is superior to the other for patients with BAV and AS, we chose Sapien XT valve as the default TAVR device for all 20 patients with BAV and AS from April 2016 to October 2017, which consists of the Sapien XT group of the patients in this study, having considered that previous studies have already demonstrated how the balloon-expandable Sapien XT valve with a better radial strength may achieve symmetric expansion of the valve and effective sealing (6–8). Three newer-generation TAVR devices, Evolut R (Medtronic Inc., Minneapolis, MN), Sapien 3 (Edwards Lifesciences, Irvine, CA), and Portico (Abbott Vascular Inc., Santa Clara, CA), were introduced in 2017. Since the procedural outcomes of newer-generation Sapien 3 valves have been considered better than those of early-generation Sapien XT valves (9–11), we chose Sapien 3 valve as the default TAVR device and performed on 36 suchlike BAV cases from October 2017 to December 2020, also the Sapien 3 group in this study.

In our institution, the default strategy for all patients was the transfemoral (TF) approach. If a TF access was not feasible because of diseased peripheral vessels, a transapical implantation would then be considered for balloon-expandable valves. Decisions were made based on pre-procedural computed tomography (CT) scan performed on all patients. All implantations were performed in a hybrid theater, and almost all patients of the study population were treated under general anesthesia. TF TAVR was conducted using percutaneous closure devices or after surgical cutdown of the femoral artery in such cases with vessel calcifications or severe obesity. Regarding the transapical approach, anterolateral mini-thoracotomy is performed in the fifth or sixth intercostal space to obtain straight access to the left ventricular apex. This is best determined by the preoperative CT scan of the chest. In most cases, after balloon valvuloplasty had been done during rapid ventricular pacing, valve deployment was performed under fluoroscopy. After TAVR, all patients were referred to the intensive care unit and monitored for at least 1 day, whereas heart rate monitoring was continued until discharge. For the purpose of platelet inhibition, aspirin (100 mg per day) was dispensed to all patients. After TAVR, an additional dose of 75 mg of clopidogrel was administered postprocedurally for 3 months in most cases. Regarding the patients with an indication for anticoagulant therapy, they received clopidogrel and warfarin or a direct oral anticoagulant without aspirin.

### Wei's Method for Valve Sizing, Positioning, and Deployment

**Figure 1:**
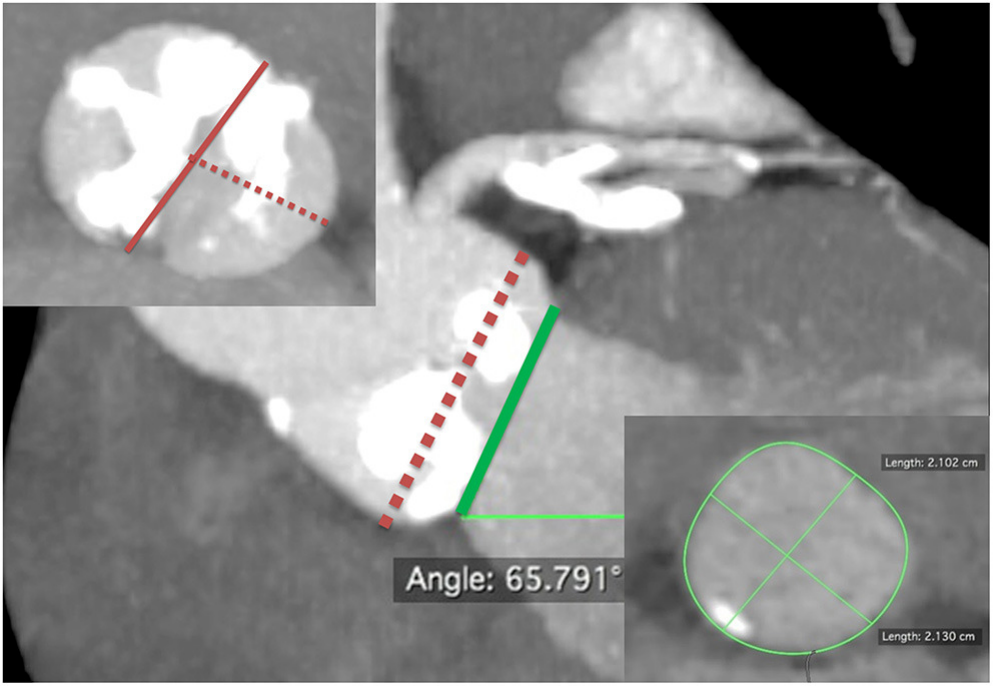
Comprehensive sizing method or the Wei's method of transcatheter aortic valve replacement for bicuspid aortic valve. First, identify a supra-annular plane at end-systole with maximum aortic leaflet opening, which predicts transcatheter heart valve (THV) prosthesis anchoring. Usually, this is at the narrowest part in the aortic valve leaflets with the most severe and asymmetric calcifications, fibrosis, or raphes, whichever makes valve anchoring feasible (central panel, red dash line). Next, measure the maximum diameter (usually, the inter-commissural distance, left upper panel, red solid line) and the minimum diameter (the shortest distance between the leading edge of the chunk of calcium/fibrosis/raphe and the opposite aortic wall, left upper panel, red dash line) at that level. Then, calculate the average diameter: (maximum diameter + minimum diameter)/2. The prosthesis is sized according to the calculated average diameter. Then, the THV is also sized in the same patient on the basis at the level of aortic annulus (central panel, green solid line) of annular area-derived diameter (conventional annular sizing method, right lower panel). If the proposed THV sizes from the two methods disagree, the prosthesis is sized according to the plane (annular or supra-annular) with the smaller derived diameter.

Currently, there is no consensus on BAV sizing, choice of THV, and/or THV implantation technique when performing TAVR in patients with BAV. This uncertainty may owe much to the different tricuspid morphological features of BAV. Nowadays, CT is the standard technique for THV sizing and procedural planning in TAVR. In addition to BAV morphological features, mentioned earlier, we have discovered from the very beginning in our series that, for BAV and AS, the THV anchoring plane is almost always supra-annular at the narrowest part of aortic valve leaflets instead of the annular level. Moreover, the presence of severe eccentric calcification can affect THV implantation and patency of the coronary ostia, so we have developed a comprehensive sizing method (the Wei's method) at our institution for patients with BAV (Figure 1).

The Wei's method is described in detail as follows:

- Identifying a supra-annular plane, which predicts THV prosthesis anchoring by scrolling the CT images in the axial view. Usually, this plane is at the narrowest part of aortic valve leaflets with the most severe and asymmetric calcifications, fibrosis, or raphes, whichever makes valve anchoring feasible.
- Measuring the longest inter-commissural distance (maximum diameter) and the shortest distance between the leading edge of the chunk of calcium/fibrosis/raphe and the opposite aortic wall at that level (minimum diameter), and then calculating the average diameter, that is, (maximum diameter + minimum diameter)/2.
- Deciding the size of prosthesis according to the calculated average diameter. A projected circle of the identical diameter to the measured average diameter is placed at that plane to simulate the apposition of the SAPIEN valve and skirt's height to the leaflets and commissures.
- Assessing the anchoring and sealing after valve expansion by taking into consideration the bulkiness of the calcium (thickness and length) and the interaction of deployed THV with calcification in the leaflets and/or raphe. Choose underfilling or overfilling of the THVs if the circle is deemed to be oversized or undersized.
- Following the supra-annular sizing, the THV is also sized in the same patient on the basis of annular area-derived diameter, which follows the conventional annular sizing method. If the proposed THV sizes from the two methods disagreed, the prosthesis is then sized according to the plane (annular or supra-annular) with the smaller derived diameter.
- Surveying the aortic root and valve anatomies to assess the risk of complications, including rupture, aortic root dissection, conduction disturbances, and coronary obstruction, and to determine the implantation depth.
- Pursuing a two-step safer implantation. That is, we deploy the THV-sized identical to the measured average diameter (anchoring), followed by post-dilatation with/without overfilling to improve conformity and reduce PVL (optimization) if needed.

### Follow-Up and Data Collection

Echocardiography and clinical follow-up were performed before and after the operation. Echocardiographic studies performed at baseline and after TAVR were evaluated according to the criteria established by the American Society of Echocardiography (30). Prediction of patient operative mortality after TAVR was calculated using the Society of Thoracic Surgeons-predicted risk of mortality (STS-PROM). All patients were followed up by the heart valve team through telephone interviews and office visits. Data were prospectively collected and entered into our heart valve replacement database.

## Definitions

Severe AS was defined as severe stenosis of the aortic valve with aortic valve area (AVA) <1.0 cm determined by transthoracic echocardiography, with or without aortic valve regurgitation. According to the Valve Academic Research Consortium-2 consensus document (31), device success was defined as (1) the absence of procedural mortality, (2) correct positioning of a single prosthetic heart valve into the proper anatomical location, and (3) intended performance of the prosthetic heart valve (no prosthesis-patient mismatch and mean aortic valve pressure gradient [PG] <20 mmHg or peak velocity <3 m/s, and no moderate or severe prosthetic valve regurgitation). Procedural success was defined as the achievement of a successful deployment of the TAVR device and retrieval of the delivery system in the absence of mortality, conversion to surgical aortic valve replacement, or myocardial infarction (MI). The implantation depth in this study was measured in the perpendicular plane of the valve, the distance of the distal part of the transcatheter heart valve to the non-coronary cusp.

The main end points of this study were the major cardiac and cerebral adverse events (MACCE), i.e., all-cause mortality, major stroke, non-fatal MI, valve failure needing reintervention, and clinically relevant valve thrombosis during long-term follow-up. Clinically relevant valve thrombosis was defined as any thrombus attached to or near an implanted THV that occludes part of the blood flow path, interferes with valve function, or is sufficiently large to warrant treatment. Other safety end points at 30 days included New York Heart Association (NYHA) functional class III/IV heart failure, life-threatening bleeding, acute kidney injury (AKI) stage 3, major vascular complications, paravalvular leaks, and the need for permanent pacer implantation for complete heart block. AKI stage 3 was defined as a change in serum creatinine (SCr) up to 72 h compared with baseline ≧3.0-fold increase in SCr or SCr ≧4.0 mg/dl (≧354 mmol/l) according to the VARC-2 criteria (30).

### Statistical Analysis

Data were transferred from the database to the Statistical Program for Social Sciences program (version 18.0 for Windows, SPSS Inc., Chicago, IL, USA). Univariate comparisons of demographic, procedural, and outcome parameters between these two groups were made. Continuous variables are expressed as mean ± SD and were compared using the Student's *t*-test or the Wilcoxon rank-sum test. Categorical variables were presented as percent frequency and compared using the Pearson's chi-square test or the Fisher's exact test.

As for the survival analysis, the patients who underwent TAVR were divided into two groups, depending on whether or not MACCE occurred during follow-up. Univariate comparisons of clinical characteristics and laboratory measurements between the two groups were conducted using appropriate tests. The independent predictors of MACCE in the patients in this study were determined using multivariate Cox proportional hazards analyses. Variables with a *p*-value < 0.1 in the univariate analysis were included in the multivariate model, in addition to the use of early- vs. newer-generation valves, and important covariables associated with poor outcome, i.e., STS-PROM score, left ventricular ejection fraction, and chronic kidney disease ≧ stage 3.

A two-sided *p* < 0.05 was considered statistically significant for all analyses. Statistical analysis was performed using SPSS version 18.0 statistical software (IBM SPSS Inc.).

## Results

### Baseline Characteristics of the Patients in This Study

Between 2016 and 2020, a total of 412 consecutive patients underwent TAVR at the Cheng Hsin General Hospital; BAV morphology was found in 56 of them (13.6%).

Baseline demographic and clinical characteristics between the Sapien XT (*n* = 20) and the Sapien 3 (*n* = 36) groups are summarized in Table 1. In general, the two groups were well matched. Although patients in the Sapien 3 group tended to have less frequently diabetes mellitus (Sapien XT vs. Sapien 3 = 45 vs. 19%, *p* = 0.085), coronary artery disease (Sapien XT vs. Sapien 3 = 70 vs. 39%, *p* = 0.051), and chronic kidney disease ≧ stage 3 (Sapien XT vs. Sapien 3 = 40 vs. 17%, *p* = 0.107), the statistical differences were non-significant. There was no significant difference in the incidence of patients in NYHA functional class III/IV at presentation; nevertheless, the STS-PROM score (Sapien XT vs. Sapien 3 = 9.01 ± 8.85 vs. 4.37 ± 3.97, *p* = 0.009) and frailty score (Sapien XT vs. Sapien 3 = 2.50 ± 1.28 vs. 1.58 ± 1.02, *p* = 0.005) were significantly lower in the Sapien 3 group. The baseline hemodynamics measured by echocardiography showed no significant differences between the two groups.

**Table 1:** Baseline characteristics of the patients in this study.

	**Sapien XT (*N* = 20)**	**Sapien 3 (*N* = 36)**	***P*-value**
Age, years	73 ± 8	70 ± 13	0.356
Male, *n* (%)	11 (55%)	21 (58%)	1
Body mass index, kg/m^2^	23.25 ± 3.22	24.17 ± 4.08	0.389
Body surface area, m^2^	1.61 ± 0.20	1.63 ± 0.15	0.676
Systemic hypertension, *n* (%)	14 (70%)	25 (69%)	1
Diabetes mellitus, *n* (%)	9 (45%)	7 (19%)	0.085
Dyslipidemia, *n* (%)	8 (40%)	15 (42%)	1
Current smoker, *n* (%)	2 (10%)	1 (3%)	0.596
Coronary artery disease, *n* (%)	14 (70%)	14 (39%)	0.051
Previous myocardial infarction, *n* (%)	4 (20%)	2 (6%)	0.221
Previous percutaneous coronary intervention, *n* (%)	8 (40%)	8 (22%)	0.270
Previous coronary artery bypass grafting, *n* (%)	0 (0%)	3 (8%)	0.479
Previous valve surgery, *n* (%)	0 (0%)	0 (0%)	-
Carotid artery disease, *n* (%)	1 (5%)	3 (8%)	1
Previous stroke, *n* (%)	4 (20%)	5 (14%)	0.828
Peripheral vascular disease, *n* (%)	3 (15%)	3 (8%)	0.747
Previous atrial fibrillation / atrial flutter, *n* (%)	4 (20%)	6 (17%)	1
Previous permanent pacemaker implantation, *n* (%)	1 (5%)	2 (6%)	1
Chronic obstructive pulmonary disease, *n* (%)	2 (10%)	2 (6%)	0.938
Chronic kidney disease ≧ stage 3, *n* (%)	8 (40%)	6 (17%)	0.107
Renal dialysis, *n* (%)	2 (10%)	1 (3%)	0.596
Heart failure, NYHA functional class III/IV, *n* (%)	17 (85%)	28 (78%)	0.764
Syncope, *n* (%)	2 (10%)	5 (14%)	1
STS-PROM score, %	9.01 ± 8.85	4.37 ± 3.97	0.009
Frailty score	2.50 ± 1.28	1.58 ± 1.02	0.005
**Baseline echocardiographic findings**			
Mean gradient, mmHg	50.15 ± 21.71	55.17 ± 24.34	0.446
Aortic valve area, cm^2^	0.61 ± 0.19	0.64 ± 0.18	0.531
Aortic regurgitation ≧ moderate, n (%)	3 (15%)	9 (25%)	0.593
Mitral regurgitation ≧ moderate, *n* (%)	6 (30%)	11 (31%)	1
Left ventricular ejection fraction, %	48.40 ± 18.44	55.17 ± 13.79	0.162
Pulmonary hypertension (PASP ≧60 mmHg), *n* (%)	3 (15%)	3 (8%)	0.747

*NYHA, New York Heart Association; STS-PROM, society for thoracic surgery-probability of mortality score; PASP, pulmonary artery systolic pressure*.

### Baseline Echocardiographic and CT Measurements of the Patients in This Study

Bicuspid valve morphology can be readily identified by CT and is commonly described, following the classification proposed by Sievers and Schmidtke (1, 2), which categorizes three main types of BAV according to the number of seam-like raphes connecting the leaflets. In this study, the frequencies of types 0, 1, and 2 morphologies of bicuspid valve were, respectively, 23/56 (41%), 30/56 (54%), and 3/56 (5%), and were well-matched between the two groups. According to another TAVR directed and simplified non-numerical classifications based on heterogeneous leaflet morphologies and leaflet orientation proposed by (16), 23/56 (41%) were classified as bicommissural non-raphe type, 30/56 (54%) as bicommissural raphe type, and 3/56 (5%) as tricommissural, respectively, in the patients in this study (Table 2).

**Table 2:** Baseline computed tomographic measurements of the patients in this study.

	**Sapien XT (*N* = 20)**	**Sapien 3 (*N* = 36)**	***P*-value**
Bicuspid morphology (Sievers classification)			
Type 0, *n* (%)	6 (30%)	17 (47%)	0.331
Type 1, *n* (%)	12 (60%)	18 (50%)	0.660
Type 2, *n* (%)	2 (10%)	1 (3%)	0.596
Bicuspid morphology (TAVR-Specific classification)			
Bicommissural non-Raphe-type, *n* (%)	6 (30%)	17 (47%)	0.331
Bicommissural Raphe-type, *n* (%)	12 (60%)	18 (50%)	0.660
Tricommissural type, *n* (%)	2 (10%)	1 (3%)	0.596
Distribution of calcium			
Calcified raphe > 4 mm, *n* (%)	5 (25%)	14 (39%)	0.449
One leaflet, *n* (%)	5 (25%)	6 (17%)	0.688
Two leaflets, *n* (%)	14 (70%)	29 (81%)	0.571
One commissure, *n* (%)	6 (30%)	11 (31%)	1
Two commissures, *n* (%)	1 (5%)	1 (3%)	1
Asymmetrical distribution of calcium, *n* (%)	16 (80%)	26 (72%)	0.747
Sino-tubular junction diameter, mm	28.95 ± 2.77	32.26 ± 4.81	0.002
Sinus of Valsalva diameter, mm	31.49 ± 3.12	32.85 ± 3.97	0.191
Left coronary height, mm	14.50 ± 3.33	15.53 ± 3.83	0.316
Right coronary height, mm	16.91 ± 3.12	18.10 ± 3.84	0.242
Porcelain aorta, *n* (%)	0 (0%)	0 (0%)	-
Aortic root angle, degree	52.65 ± 8.44	55.61 ± 10.42	0.282
Ascending aorta, 3 cm above the annulus, mm	39.03 ± 4.25	43.74 ± 6.71	0.002
Aortopathy (aortic diameter > 4.5 cm), *n* (%)	3 (15%)	17 (47%)	0.034

*TAVR, transcatheter aortic valve replacement*.

Moreover, CT assessment also showed that eccentric calcification was common in BAV and present in, respectively, 16/20 (80%) and 26/36 (72%) patients who underwent TAVR with Sapien XT and Sapien 3. There were 5/20 (25%) patients in the Sapien XT group and 14/36 (39%) in the Sapien 3 with a calcified raphe >4 mm present. The distribution of calcium was seen on two leaflets in 43/56 (77%), one commissure in 17/56 (30%), one leaflet in 11/56 (20%), and two commissures in 2/56 (4%) patients of the study population. Regarding the aortic root and ascending aorta anatomies, the coronary heights and aortic root angles were similar in both groups. But the patients in the Sapien 3 group had significantly larger sino-tubular junctions (Sapien XT vs. Sapien 3 = 28.95 ± 2.77 vs. 32.26 ± 4.81, *p* = 0.002), ascending aorta dimensions (Sapien XT vs. Sapien 3 = 39.03 ± 4.25 vs. 43.74 ± 6.71, *p* = 0.002), and more aortopathy (Sapien XT vs. Sapien 3 = 15% vs. 47%, *p* = 0.034), compared to those patients in the Sapien XT group.

### Transcatheter Heart Valve Sizes Proposed by Annular vs. Supra-Annular Sizing Methods of the Study Populations

As shown in Table 3, the valve sizes ranged from 23 to 29 mm for the Sapien XT and Sapien 3 devices in both groups. The most commonly used valve sizes were 23 mm (45%) and 26 mm (45%) in the Sapien XT group, and 23 mm (39%), and 26 mm (33%) in the Sapien 3 group. The mean area-derived diameter and supra-annular sizing diameter were similar in both the Sapien XT and Sapien 3 groups. However, when the aforementioned valve sizing criteria were applied, there existed 11/56 (20%) discrepancies in the proposed THV size between the conventional valve sizing and supra-annular sizing methods. Compared with annular sizing, supra-annular sizing resulted in 45/56 (80%) similar sizes, 7/56 (13%) larger sizes, and 4/56 (7%) smaller sizes. Furthermore, a smaller valve was selected in the Sapien 3 cases compared to the Sapien XT cases, and the percentages of annular area oversizing were 2.89 ± 7.69 vs. 7.26 ± 4.44% (*p* = 0.009) as measured by the conventional annular sizing method, and 2.08 ± 5.56 vs. 5.77 ± 4.98% (*P* = 0.017) by supra-annular sizing method.

**Table 3:** Transcatheter heart valve size and valve sizes proposed by different sizing methods of the patients in this study.

	**Sapien XT (*N* = 20)**	**Sapien 3 (*N* = 36)**	***P*-value**
**Transcatheter heart valve size, mm**			
20, *n* (%)	0 (0%)	0 (0%)	-
23, *n* (%)	9 (45%)	16 (44%)	1
26, *n* (%)	8 (40%)	16 (44%)	0.968
29, *n* (%)	3 (15%)	4 (12%)	1
**Conventional annular sizing method**			
Maximum diameter, mm	26.35 ± 2.85	27.36 ± 3.56	0.281
Minimum diameter, mm	20.67 ± 2.02	21.77 ± 2.59	0.106
Mean diameter, mm	23.52 ± 2.17	24.54 ± 2.84	0.167
Perimeter-derived diameter, mm	23.83 ± 2.21	24.89 ± 3.04	0.175
Area-derived diameter, mm	23.43 ± 2.15	24.45 ± 2.91	0.176
Proposed valve size, mm			
20, *n* (%)	0 (0%)	1 (3%)	1
23, *n* (%)	9 (45%)	14 (39%)	0.871
26, *n* (%)	9 (45%)	12 (33%)	0.565
29, *n* (%)	2 (10%)	9 (25%)	0.316
Oversizing, %	7.26 ± 4.44	2.89 ± 7.69	0.009
**Supra-annular sizing (The Wei's method)**			
Maximum diameter, mm	27.43 ± 2.59	28.10 ± 3.05	0.406
Minimum diameter, mm	20.08 ± 2.98	20.94 ± 2.88	0.296
Mean diameter, mm	23.75 ± 2.07	24.56 ± 2.40	0.206
Proposed valve size, mm			
20, *n* (%)	0 (0%)	0 (0%)	-
23, *n* (%)	9 (45%)	14 (39%)	0.871
26, *n* (%)	9 (45%)	18 (50%)	0.936
29, *n* (%)	2 (10%)	4 (11%)	1
Oversizing, %	5.77 ± 4.98	2.08 ± 5.56	0.017
**Discordance of sizing (Annular vs. supra-annular)**, ***n*** **(%)**	4 (20%)	7 (19%)	1
Smaller, *n* (%)	2 (10%)	5 (14%)	1
Larger, *n* (%)	2 (10%)	2 (6%)	0.938

### Procedural Characteristics and Immediate Complications

The technical aspects of the procedure and procedural outcomes are presented in Table 4. TAVR procedures were conducted *via* TF in 19 (95%) Sapien XT cases and 35 (97%) Sapien 3 cases. The Sapien XT and Sapien 3 valves were, respectively implanted *via* transapical access in one (5%) and one (3%) of the patients in this study. Besides, Sapien 3 was more frequently implanted with requirement for balloon valvuloplasty for post-dilatation (Sapien XT 35% vs. Sapien 3 89%, *p* < 0.001) rather than pre-dilatation (100% pre-dilatation before Sapien XT and 81% before Sapien 3, *p* = 0.092). The final implantation depth below the annulus was similar in both.

**Table 4:** Procedural characteristics and immediate complications of the patients in this study.

	**Sapien XT (*N* = 20)**	**Sapien 3 (*N* = 36)**	***P*-value**
Vascular access			
Trans-femoral, *n* (%)	19 (95%)	35 (97%)	1
Trans-apical, *n* (%)	1 (5%)	1 (3%)	1
Pre-dilatation, *n* (%)	20 (100%)	29 (81%)	0.092
Post-dilation, *n* (%)	7 (35%)	32 (89%)	<0.001
Implantation depth from annulus, mm	2.20 ± 1.60	2.36 ± 1.25	0.677
Device success, *n* (%)	17 (85%)	34 (94%)	0.485
Paravalvular leakage ≧ moderate, *n* (%)	2 (10%)	1 (3%)	0.596
2nd device needed, *n* (%)	0 (0%)	0 (0%)	-
Post-TAVR trans-valvular PG ≧ 20 mmHg, *n* (%)	0 (0%)	1 (3%)	1
Procedural success, *n* (%)	19 (95%)	36 (100%)	0.764
Conversion to SAVR, *n* (%)	0 (0%)	0 (0%)	-
Coronary obstruction, *n* (%)	2 (10%)	0 (0%)	0.238
Annulus rupture, *n* (%)	0 (0%)	0 (0%)	-
Left ventricular rupture, *n* (%)	0 (0%)	0 (0%)	-
Emergency CPB/ECMO, *n* (%)	1 (5%)	0 (0%)	0.764
Total procedure time, min	38.70 ± 25.54	31.11 ± 12.14	0.137
Total fluoroscopic time, min	22.93 ± 12.36	19.25 ± 7.15	0.163
Total contrast volume, mL	148.55 ± 56.20	99.97 ± 28.27	0.001

*TAVR, transcatheter aortic valve replacement; PG, pressure gradient; SAVR, surgical aortic valve replacement; CPB/ECMO, cardiopulmonary bypass/extracorporeal membrane oxygenation*.

None of the 56 patients in this study required implantation of a second valve due to an initial implant embolization or malpositioning. Significant PVL (≧ moderate degree) after the TAVR procedure was found in two (10%) patients with Sapien XT and one (3%) with Sapien 3, respectively (*p* = 0.596). One (3%) patient in the Sapien 3 group had a post-procedural trans-valvular gradient of >20 mmHg. To sum up, the device success rates were 85% for Sapien XT and 94% for Sapien 3 (*p* = 0.485).

Major intraoperative complications like emergency conversion to surgical aortic valve replacement, and annular or left ventricular rupture did not happen in either group. Two (10%) patients suffered from acute coronary occlusion and were successfully treated with percutaneous coronary intervention and stenting, although one of them needed emergent hemodynamic support. The procedural success rates were 95% for Sapien XT and 100% for Sapien 3 (*p* = 0.764). The mean procedure and fluoroscopic times of the two groups were similar; however, the Sapien 3 group received significantly less contrast volume (Sapien XT 148.55 ± 56.20 ml vs. Sapien 3 99.97 ± 28.27 ml; *p* = 0.001).

### Thirty-Day Hemodynamic Performance of the THV and Clinical Outcomes

Transcatheter valve performance was determined by echocardiography at the 30-day follow-up (Table 5). A significant reduction in prosthetic valvular PG and an increase in prosthetic AVAs at 30 days were observed in all patients who underwent TAVR successfully. However, a trend toward higher mean trans-aortic valve PG (Sapien XT vs. Sapien 3 = 8.69 ± 3.05 mmHg vs. 11.03 ± 5.04 mmHg, *p* = 0.066) and smaller AVA (Sapien XT vs. Sapien 3 = 1.97 ± 0.35 cm^2^ vs. 1.82 ± 0.25 cm^2^, *p* = 0.089) was also observed in patients who underwent TAVR with Sapien 3, although these were not statistically significant. Echocardiography follow-up showed no significant difference in left ventricular ejection fraction and pulmonary artery systolic pressure of the two groups. Moderate/severe aortic regurgitation incidence was not statistically different between the two devices (Sapien XT vs. Sapien 3 = 10 vs. 6%, *p* = 0.938).

**Table 5:** Thirty-day hemodynamic performance of the THV and 30-day and long-term clinical outcomes of the patients in this study.

	**Sapien XT (*N* = 20)**	**Sapien 3 (*N* = 36)**	***P*-value**
Intensive care unit stay, days	2.85 ± 4.61	1.17 ± 0.45	0.120
30-day NYHA functional class			
III/IV, *n* (%)	4 (20%)	1 (3%)	0.094
30-day MACCE, *n* (%)	3 (15%)	3 (8%)	0.747
All-cause mortality, *n* (%)	0 (0%)	0 (0%)	-
Cardiac mortality, *n* (%)	0 (0%)	0 (0%)	-
Non-fatal myocardial infarction, *n* (%)	0 (0%)	0 (0%)	-
Non-fatal stroke, *n* (%)	1 (5%)	0 (0%)	0.764
Other 30-day VARC-2 complications			
Major vascular access complication, *n* (%)	0 (0%)	0 (0%)	-
Acute kidney injury, stage 3, *n* (%)	0 (0%)	0 (0%)	-
Permanent pacemaker implantation for CAVB, *n* (%)	2 (10%)	2 (6%)	0.938
Hemodynamics by echocardiography at 30-day			
Mean gradient, mmHg	8.69 ± 3.05	11.03 ± 5.04	0.066
Aortic valve area, cm^2^	1.97 ± 0.35	1.82 ± 0.25	0.089
Aortic regurgitation ≧ moderate, *n* (%)	2 (10%)	2 (6%)	0.938
Left ventricular ejection fraction, %	55.70 ± 13.55	58.30 ± 11.13	0.451
Pulmonary hypertension (PASP ≧60 mmHg), *n* (%)	1 (5%)	2 (6%)	1
Long-term cumulative MACCE, *n* (%)	7 (35%)	4 (11%)	0.071
All-cause mortality, *n* (%)	3 (15%)	0 (0%)	0.077
Cardiac mortality, *n* (%)	0 (0%)	0 (0%)	-
Non-fatal myocardial infarction, *n* (%)	0 (0%)	1 (3%)	1
Non-fatal stroke, *n* (%)	2 (10%)	1 (3%)	0.596
Valve failure, *n* (%)	1 (5%)	1 (3%)	1
Clinically relevant Valve thrombus, *n* (%)	1 (5%)	1 (3%)	1

*THV, transcatheter heart valve; NYHA, New York Heart Association; MACCE, major adverse cardiac cerebral events; VARC, valve academic research consortium; CAVB, complete atrioventricular block; PASP, pulmonary artery systolic pressure*.

The intensive care unit stays were similar between the two groups. Significant improvement in NYHA functional class was observed in both groups. At 30 days, there were no all-cause mortality, cardiovascular mortality, non-fatal MI, major or life-threatening bleeding, AKI stage 3, or major vascular complications in either, though one patient in the Sapien XT group suffered from nonfatal stroke. The rates of needing a permanent pacemaker were similar in both groups (Sapien XT vs. Sapien 3 = 10 vs. 6%, *p* = 0.938).

During a median follow-up of 743 days (interquartile range: 393–1016 days), the long-term clinical outcomes of the newer-generation Sapien 3 group were better than those of the early-generation Sapien XT (MACCE rates 35 vs. 11%, *P* = 0.071). One patient in either group experienced clinically relevant valve thrombosis needing anticoagulant therapy. Valve failure needing reintervention was reported in one (5%) patient in the Sapien XT group and one (3%) in the Sapien 3 group.

**Figure 2:**
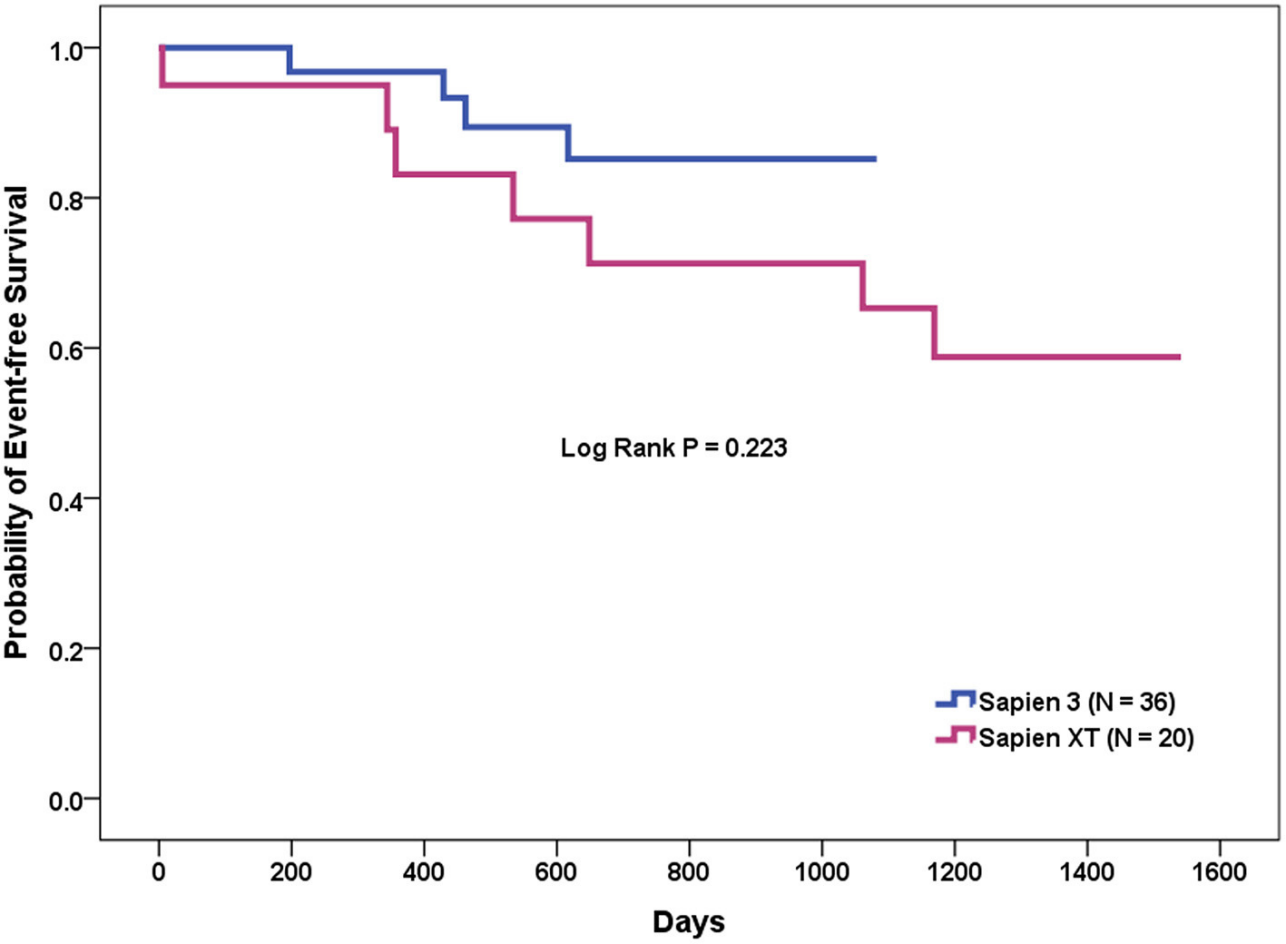
Event-free survival curve of transcatheter aortic valve replacement for bicuspid aortic valve stenosis with the Sapien XT vs. Sapien 3 devices.

The patients who underwent TAVR were further divided into two groups, depending on whether or not MACCE occurred during follow-up (Table 6). In the Cox proportional hazards analyses, the presence of a calcified raphe > 4 mm (*p* = 0.032), lower-left coronary height (*p* = 0.045), and the use of Sapien 3 device (*p* = 0.041) are significant predictors of MACCE according to the univariate analysis. The Kaplan-Meier analysis showed that the event-free survival rate was better in those patients who underwent TAVR with newer-generation Sapien 3 valves, but the statistical differences were non-significant (log-rank test, *p* = 0.223) (Figure 2). However, further multivariate analyses, using variables that included device types, important covariables associated with poor outcome, that is, STS-PROM score, left ventricular ejection fraction and chronic renal failure, and those variables associated with the MACCE in the univariate analysis, identified the presence of calcified raphe > 4 mm as the only independent predictor of long-term MACCE (hazard ratio: 6.76; 95% confidence interval: 1.21–37.67, *p* = 0.029). One such patient with Sapien 3 implantation needed percutaneous PVL repair following TAVR due to the development of refractory heart failure 3 months after TAVR procedure.

**Table 6:** Independent prognostic determinants of long-term composite MACCE by univariate and multivariate analyses.

	**MACCE (+) (*N* = 11)**	**MACCE (-) (*N* = 45)**	**Univariate *P*-value**	**Multivariate *P*-value**
Baseline characteristics				
Age, years	71 ± 7	71 ± 13	0.912	
Male, *n* (%)	8 (73%)	24 (53%)	0.409	
Body mass index, kg/m^2^	22.42 ± 2.91	24.19 ± 3.93	0.166	
Body surface area, m^2^	1.66 ± 0.17	1.62 ± 0.17	0.481	
Systemic hypertension, *n* (%)	8 (73%)	31 (69%)	1	
Diabetes mellitus, *n* (%)	5 (46%)	11 (24%)	0.312	
Dyslipidemia, *n* (%)	7 (64%)	16 (36%)	0.175	
Current smoker, *n* (%)	1 (9%)	2 (4%)	1	
Coronary artery disease, *n* (%)	6 (55%)	22 (49%)	1	
Previous myocardial infarction, *n* (%)	2 (18%)	4 (9%)	0.727	
Previous percutaneous coronary intervention, *n* (%)	5 (46%)	11 (24%)	0.312	
Previous coronary artery bypass grafting, *n* (%)	1 (9%)	2 (4%)	1	
Previous valve surgery, *n* (%)	0 (0%)	0 (0%)	-	
Carotid artery disease, *n* (%)	0 (0%)	4 (9%)	0.709	
Previous stroke, *n* (%)	2 (18%)	7 (16%)	1	
Peripheral vascular disease, *n* (%)	1 (9%)	5 (11%)	1	
Previous atrial fibrillation / atrial flutter, *n* (%)	4 (36%)	6 (13%)	0.177	
Previous permanent pacemaker implantation, *n* (%)	0 (0%)	3 (7%)	0.894	
Chronic obstructive pulmonary disease, *n* (%)	2 (18%)	2 (4%)	0.351	
Chronic kidney disease ≧ stage 3, *n* (%)	5 (46%)	9 (20%)	0.174	0.159
Renal dialysis, *n* (%)	1 (9%)	2 (4%)	1	
Heart failure, NYHA functional class III/IV, *n* (%)	9 (82%)	36 (80%)	1	
Syncope, *n* (%)	1 (9%)	6 (13%)	1	
STS-PROM score, %	8.39 ± 10.07	5.45± 5.27	0.180	0.814
Frailty score	2.36 ± 1.36	1.80 ± 1.14	0.163	
Baseline echocardiographic findings				
Mean gradient, mmHg	45.18 ± 15.18	55.38 ± 24.67	0.197	
Aortic valve area, cm^2^	0.64 ± 0.17	0.63 ± 0.18	0.932	
Aortic regurgitation ≧ moderate, *n* (%)	4 (36%)	8 (18%)	0.349	
Mitral regurgitation ≧ moderate, *n* (%)	3 (27%)	14 (31%)	1	
Left ventricular ejection fraction, %	45.36 ± 18.98	54.56 ± 14.58	0.083	0.713
Pulmonary hypertension (PASP ≧ 60 mmHg), *n* (%)	2 (18%)	4 (9%)	0.727	
Bicuspid morphology (Sievers classification)				
Type 0, *n* (%)	2 (18%)	21 (47%)	0.168	
Type 1, *n* (%)	8 (73%)	22 (49%)	0.278	
Type 2, *n* (%)	1 (9%)	2 (4%)	1	
Bicuspid morphology (TAVR-Specific classification)				
Bicommissural non-Raphe-type, *n* (%)	2 (18%)	21 (47%)	0.168	
Bicommissural Raphe-type, *n* (%)	8 (73%)	22 (49%)	0.278	
Tricommissural type, *n* (%)	1 (9%)	2 (4%)	1	
Distribution of calcium				
Calcified raphe >4 mm, *n* (%)	7 (64%)	12 (27%)	0.032	0.029
One leaflet, *n* (%)	1 (9%)	10 (22%)	0.576	
Two leaflets, *n* (%)	9 (82%)	34 (76%)	0.966	
One commissure, *n* (%)	4 (36%)	13 (29%)	0.906	
Two commissures, *n* (%)	1 (9%)	1 (2%)	0.846	
Asymmetrical distribution of calcium, *n* (%)	9 (82%)	33 (73%)	0.846	
Sino-tubular junction diameter, mm	29.55 ± 3.76	31.45 ± 4.59	0.208	
Sinus of Valsalva diameter, mm	31.31 ± 3.17	32.62 ± 3.83	0.299	
Left coronary height, mm	13.70 ± 2.15	15.52 ± 3.88	0.045	0.314
Right coronary height, mm	16.84 ± 2.59	17.88 ± 3.82	0.396	
Porcelain aorta, *n* (%)	0 (0%)	0 (0%)	-	
Aortic root angle, degree	51.27 ± 7.50	55.36 ± 10.18	0.218	
Ascending aorta, 3 cm above the annulus, mm	39.37 ± 6.20	42.72 ± 6.26	0.118	
Aortopathy (aortic diameter >4.5 cm), *n* (%)	2 (18%)	18 (40%)	0.316	
Transcatheter heart valve type				
≦23 mm, *n* (%)	6 (55%)	19 (42%)	0.690	
≧26 mm, *n* (%)	5 (45%)	26 (58%)	0.690	
Procedural characteristics				
Device type (Sapien 3), *n* (%)	4 (36%)	32 (71%)	0.041	0.176
Vascular access				
Trans-femoral access, *n* (%)	11 (100%)	43 (96%)	1	
Pre-dilatation, *n* (%)	11 (100%)	38 (84%)	0.374	
Post-dilatation, *n* (%)	7 (64%)	32 (71%)	0.906	
Implantation depth from annulus, mm	2.18 ± 0.85	2.33 ± 1.48	0.746	
Device success, *n* (%)	9 (82%)	42 (93%)	0.541	
Procedural success, *n* (%)	11 (100%)	44 (98%)	1	
30-day VARC complications				
Major vascular access complication, *n* (%)	0 (0%)	0 (0%)	-	
Acute kidney injury, stage 3, *n* (%)	0 (0%)	0 (0%)	-	
Permanent pacemaker implantation for CAVB, *n* (%)	1 (9%)	3 (7%)	1	
30-day NYHA functional class III/IV, *n* (%)	2 (18%)	3 (7%)	0.541	
Hemodynamics by echocardiography at 30-day				
Mean gradient, mmHg	8.91 ± 2.84	1.47 ± 4.83	0.312	
Aortic valve area, cm^2^	1.89 ± 0.33	1.87 ± 0.29	0.801	
Aortic regurgitation ≧ moderate, *n* (%)	2 (18%)	2 (4%)	0.351	
Left ventricular ejection fraction, %	52.55 ± 13.85	58.57 ± 11.37	0.141	
Pulmonary hypertension (PASP ≧ 60 mmHg), *n* (%)	0 (0%)	3 (7%)	0.894	

*MACCE, major adverse cardiac cerebral events; NYHA, New York Heart Association; STS-PROM, society for thoracic surgery-probability of mortality score; PASP, pulmonary artery systolic pressure; TAVR, transcatheter aortic valve replacement; VARC, valve academic research consortium; CAVB, complete atrioventricular block*.

## Discussion

The main findings of our study are as follows: (1) to the best of our knowledge, this is the first report of the prevalence of BAV in patients with critical AS referred for TAVR in Taiwan; (2) the use of newer-generation balloon-expandable Sapien 3 valve may achieve better TAVR outcomes in patients with BAV compared to the early-generation Sapien XT valve; however, the benefit of reducing PVL due to the outer skirt of Sapien 3 may be accompanied by a tradeoff of reduced effective orifice area (EOA); (3) the complementary approach of supra-annular sizing to conventional annular sizing method (Wei's Method) developed by our team is useful in providing alternative guidance to perform safer THV implantation; and (4) the presence of calcified raphe > 4 mm was the only independent predictor of long-term outcomes in the present study so percutaneous PVL repair following TAVR in certain patients may be needed.

In our series, between 2016 and 2020, BAV morphology was found in 56/412 (13.6%) consecutive patients who underwent TAVR at the Cheng Hsin General Hospital, which was roughly 10% comparable to those reported from other Asian patient populations referred to TAVR (7, 32). Regarding the bicuspid valve morphology, according to the classification proposed by Sievers and Schmidtke, the frequencies of types 0, 1, and 2 morphologies of bicuspid valve were, respectively, 39, 55, and 5%. According to other simplified non-numerical classifications proposed by (16) 41, 54, and 5% of BAV in the patients in this study were classified, respectively, as bicommissural non-raphe type, bicommissural raphe type, and tricommissural. These were also roughly comparable to those reported by others (8–15).

Traditionally, surgical aortic valve replacement is performed to treat BAV with AS and/or aortic regurgitation (1, 5). The new American guidelines also recommend TAVR for BAV to be performed only in selected patients with BAV to address the concerns regarding the procedural and device success rates and long-term durability of THVs, particularly in the younger BAV population (5). However, since the indication of TAVR has been extended to younger low-risk patients with critical AS (5), the proportion of patients with BAV undergoing TAVR is likely to increase. TAVR originally developed for tricuspid AS has been applied to patients with BAV as an off-label indication, and there is a growing interest in the safety and efficiency of TAVR in these patients. The studies on TAVR with early-generation THVs have highlighted the complexity of performing the procedure in patients with BAV, with high rates of malposition, the need for multiple THVs, and relatively high rates of moderate-to-severe residual PVL (6–8). More recently, data from large registries demonstrated that the use of newer-generation devices featuring repositionability, sealing properties, and more accurate deployment yielded better outcomes than the early-generation devices had ever done in patients with BAV (9–15). However, complications such as moderate or severe PVL and aortic root dissection are more commonly seen in patients with BAV compared to those in patients with tricuspid aortic valve. Moreover, a clear-cut answer regarding whether newer-generation Sapien 3 valve is better than the early-generation Sapien XT valve for BAV or not has yet to be sought. In this study, we demonstrated that the use of newer-generation balloon-expandable Sapien 3 valve achieved better TAVR outcomes in patients with BAV compared to the early-generation Sapien XT valve, though it is considered statistically insignificant. The outer fabric seal of Sapien 3 did adapt better to the irregular annuli shapes and the asymmetrically calcified leaflets in patients with BAV; thus, compared with the Sapien XT group, the Sapien 3 group demonstrated numerical lower rates of ≧ moderate PVL (10 vs. 3%, *p* = 0.596), even though the CT oversizing percentage values were significantly lower in the Sapien 3 vs. Sapien XT groups (percentages of annular area oversizing were 2.89 ± 7.69% vs. 7.26 ± 4.44%, *p* = 0.009 as measured by the conventional annular sizing method and 2.08 ± 5.56% vs. 5.77 ± 4.98%, *p* = 0.017 by the supra-annular sizing method, respectively). Nevertheless, it is worth noting that, at 30 days, follow-up echocardiography showed that the EOA was smaller (1.82 ± 0.25 vs. 1.97 ± 0.35 cm^2^; *p* = 0.089) and the mean trans-valvular PG higher (11.03 ± 5.04 vs. 8.69 ± 3.05 mmHg; *p* = 0.066) in Sapien 3 vs. Sapien XT, though again it is considered statistically insignificant. These findings are in line with those of previous reports, that is, the benefit of reducing PVL due to the outer skirt of Sapien 3 may be accompanied by a tradeoff of reduced EOA (33, 34). Although a smaller EOA is unlikely to affect short-term clinical outcomes, whether it may give rise to hemodynamic alterations and has a negative effect on valve durability still needs a longer-term follow-up investigation.

Some experts have proposed various supra-annular sizing methods, algorithms, balloon sizing, or even computer simulation to improve valve sizing and device selection, hoping to reduce complications (12, 16–26). Whether supra-annular sizing can truly provide additional benefits in terms of improving device and procedural success and/or clinical outcomes remains controversial (27–29) because the supra-annular sizing is less reproducible than annular sizing, and its techniques of measurements not yet standardized (17–26). However, as shown in this study, although there was no clinically significant difference between annular and supra-annular sizings, supra-annular sizing, which selects a smaller THV than suggested by annular sizing and thus avoids the oversizing-related risks for the minority of patients with tapered or funnel anatomy, appeared to be of incremental value. Suchlike cases as discussed here consisted of four out of the 56 (7%) of the patients in our series. Compared to the use of the circle method for supra-annular sizing, advocated in BAV cases by a Bicuspid Expert Panel of interventional cardiologists and cardiac surgeons (26), moreover, our method is much easier to apply and time-saving, and the diameter of THV derived is more precise and may guarantee safer implantation.

Regarding the procedural characteristics, pre-dilatation of the BAV is performed in TAVR more frequently with Sapien XT than with Sapien 3 valve (100 vs. 81%, *p* = 0.092) to facilitate the crossing of the delivery system and ensure appropriate expansion of the THVs. However, balloon pre-dilatation with contrast injection is also used often to observe the behavior of leaflets in relation to coronary ostia and aortic wall because of the presence of a heavy and asymmetrical distribution of calcium; and even with the use of Sapien 3, the risks of annular or aortic rupture and coronary obstruction are not entirely avoidable. On the other hand, post-dilatation is performed in TAVR more frequently with Sapien 3 than with Sapien XT (89 vs. 35%, *p* < 0.001), a much higher frequency than reported in the published data on the Sapien 3 (9–15). This may be owing to the less aggressive oversizing of the Sapien 3 compared to that of the Sapien XT and more patients in the Sapien 3 group needed post-dilatation with/without overfilling to improve conformity and reduce PVL (optimization) in our series. During TAVR procedures, two (10%) patients suffered from acute coronary occlusion and were successfully treated with percutaneous coronary intervention and stenting, although one of them needed emergent hemodynamic support. Although we use coronary protection technique whenever coronary obstruction is anticipated on pre-procedural CT, these two events were not the case. Regarding the relatively high rates of moderate to severe PVL after TAVR procedure in two (10%) patients with Sapien XT and one (3%) with Sapien 3, they all resulted from the presence of severe calcification of the raphe or a bulky calcium on one cusp, instead of the undersizing THVs. One such patient with Sapien 3 implantation needed percutaneous PVL repair following TAVR due to the development of refractory heart failure 3 months after TAVR procedure.

At 30 days, there was no mortality, non-fatal MI, major bleeding, nor vascular complications or significant differences in the incidences of stroke and AKI stage 3, or rates of need for a permanent pacemaker in either group. During a median follow-up of 743 days, the long-term clinical outcomes of newer-generation Sapien 3 were better than those of early-generation Sapien XT, though it was statistically non-significant (MACCE rates 35 vs. 11%, *p* = 0.071). The presence of a calcified raphe >4 mm, lower-left coronary height, and the use of Sapien XT device are significant predictors of MACCE according to univariate analysis; nevertheless, multivariate analysis identified the presence of a calcified raphe >4 mm as the only independent predictor of long-term MACCE (hazard ratio: 6.76; 95% confidence interval: 1.21–37.67, *p* = 0.029) after adjustment of device types, important covariables associated with poor outcome, and those variables associated with MACCE in the univariate analysis. In other words, although the evolution in patient selection, valve sizing, choice of THV, and procedural characteristics may affect clinical outcomes of patients with BAV undergoing TAVR over time, our results suggested that the most important factor in determining device success and long-term outcomes is the presence of unfavorable aortic and leaflets anatomies; in particular, a calcified raphe. As we already know, BAVs are more heavily calcified than tricuspid aortic valve and the calcification burden is more eccentric and asymmetrical as demonstrated in our study and others' (12–16). The presence of a calcified raphe and the heterogeneous distribution of the calcium of the BAV may prevent optimal expansion of the THV stent frame, resulting in elliptical implantation, malapposition, migration, and significant PVL. According to a recently published study by Yoon et al. (15) patients with combined calcified raphe and excessive leaflet calcium were of the highest risk phenotype associated with more frequent procedural complications like aortic root injury and PVL, and a 3-fold higher mortality, which is inconsistent with our findings. Therefore, in younger patients with unfavorable BAV anatomies and at low operative risk, the best strategy at this stage probably is a referral for surgical aortic valve replacement since the outcomes of surgery are excellent (1, 5). Moreover, the data concerning the outcomes of surgical aortic valve replacement in elderly patients with BAV at increased surgical risk are lacking. For these patients, less invasive approaches like intravascular lithotripsy are called for. The first-in-man report of intravascular lithotripsy is promising, but further studies are needed to confirm the safety and feasibility of its use in TAVR (34).

Finally, this study used only balloon-expandable valves; although previous studies of TAVR for BAV demonstrated that no difference existed in short- and mid-term TAVR outcomes with balloon-expandable valves and self-expanding valves, balloon-expandable valves still presented a higher risk of annular rupture in comparison with self-expanding valve, although it never happened to the patients in this study (9–15). Actually, the individual heart team's preferences decide what device types to choose, and the newer-generation devices may produce the same outcomes. In the future, specifically designed prospective studies are required to provide further evidence of anatomical selection criteria, durability, and long-term success rates of different devices before TAVR can really be deemed to be a viable option for all younger patients with BAV.

### Study Limitations

Considering small number of patients in both groups and the fact that it was not a multicenter study, the results reported here, particularly concerning the comparisons between the two THVs, should be treated with caution. Secondly, although the two prosthesis groups were similar in terms of comorbidities and pre-procedural risk, our study was not a randomized trial and, hence, subjected to selection bias and unmeasured confounders; no definite conclusions can be drawn. Thirdly, two different TAVR devices were implanted across a long time frame of 4 years from 2016 to 2020. During that period, TAVR for the treatment of BAV with AS has evolved drastically. With the cumulating experiences of our heart team and the continuous technical refinements of the devices and delivery systems, a shift toward treating lower-risk patients who underwent TAVR has been taking place and is perhaps associated with a survival benefit in the patients in this study.

## Conclusion

The results reported herein are of the largest series of TAVR for BAV with the use of balloon-expandable Sapien XT and Sapien 3 valves in Taiwan. We found that BAV anatomy, especially the presence of a calcified raphe and associated technical challenges for the TAVR procedure, is the most important determinant of procedural and clinical outcomes. Since patients with BAV are usually younger, with longer life expectancy, and perhaps need one or more interventions during the rest of their lives, we naturally expect the best possible results of the index procedure through optimal patient selection, anatomical consideration, and procedural planning in order to guarantee satisfactory long-term outcomes.

## Data Availability Statement

The raw data supporting the conclusions of this article will be made available by the authors, without undue reservation.

## Ethics Statement

The studies involving human participants were reviewed and approved by Institutional Review Board of Cheng Hsin General Hospital. Written informed consent for participation was not required for this study in accordance with the national legislation and the institutional requirements.

## Author Contributions

W-HY, Y-TL, and JW conceived of the presented idea, developed the theory, performed the computations, analyzed and interpreted data, and verified the analytical methods. W-HY, Y-TL, T-PT, K-CL, M-CH, and JW provided the patients in this study and performed the TAVR procedures. W-HY, Y-TL, Y-HT, and JW collected and assembled data. W-HY and JW supervised the findings of this work. All authors discussed the results and contributed to the final manuscript and agree to be accountable for the content of the work.

## Funding

The study was funded by the Cheng Hsin General Hospital grant number CHGH109-(IP)4.

## Conflict of Interest

The authors declare that the research was conducted in the absence of any commercial or financial relationships that could be construed as a potential conflict of interest.

## Publisher's Note

All claims expressed in this article are solely those of the authors and do not necessarily represent those of their affiliated organizations, or those of the publisher, the editors and the reviewers. Any product that may be evaluated in this article, or claim that may be made by its manufacturer, is not guaranteed or endorsed by the publisher.
